# Personalized Deep Networks for Enhanced ECG Segmentation

**DOI:** 10.1007/s13239-026-00835-z

**Published:** 2026-04-20

**Authors:** Maylon Pereira Folli, Gabriel Tozatto Zago, Stephanie Rezende Alvarenga Moulin Mares, Rodrigo Varejão Andreão

**Affiliations:** 1 Electric Engineering Department, Federal Institute of Espírito Santo, Avenida Vitória, Vitória, 29040-780 Espírito Santo Brazil; 2 Automation and Control Engineering Department, Federal Institute of Espírito Santo, Avenida dos Sabiás, Serra, 29166-630 Espírito Santo Brazil; 3 MD, Hospital Naval de Brasília, SEPS Q 711/911 - Asa Sul, Brasília, 70390-115 Distrito Federal Brazil

**Keywords:** Electrocardiography (ECG), Deep learning, Patient-specific adaptation, Neural networks, ECG segmentation, Convolutional neural networks (CNNs), Long short-term memory (LSTM), QT database, Biomedical signal processing, Personalized healthcare

## Abstract

Electrocardiography (ECG) plays a vital role in the diagnosis of cardiovascular diseases by analyzing the electrical activity of the heart. ECG semantic segmentation is a subfield focused on sample-wise delineation of ECG waveforms by assigning a physiological label to each time sample, enabling explicit estimation of clinically meaningful onset and offset boundaries. Recent advancements in deep learning have significantly improved ECG classification accuracy; however, the same has not yet been observed in automatic ECG segmentation. Existing models often lack explainability and adaptability to patient-specific variations, thereby reducing their generalizability. This study proposes a personalized deep neural network approach for enhanced ECG processing. This method incorporates convolutional neural networks (CNNs) and bidirectional long short-term memory (BiLSTM) networks, incorporating an attention mechanism to refine segmentation accuracy. A novel loss function is introduced to ensure smoother temporal transitions and better classification accuracy. The model was evaluated using the QT Database, demonstrating substantial improvements in P-wave and QRS delineation and in T-wave offset localization segmentation when fine-tuned for individual patients when fine-tuned and evaluated on held-out data from the same patient, demonstrating the benefit of intra-patient adaptation. Our results indicate that personalization improves delineation accuracy for challenging waveforms (notably P and T waves), supporting the potential of deep learning to better capture patient-specific morphology and providing a stronger basis for waveform-level, clinically interpretable ECG analysis.

## Introduction

Electrocardiography (ECG) plays a crucial role in the diagnosis and management of cardiovascular diseases, which remain the leading cause of morbidity and mortality worldwide [1, 2]. As a noninvasive tool, ECG provides real-time insights into the electrical activity of the heart, making it indispensable to detect arrhythmias, myocardial ischemia, and other cardiac abnormalities. Its ability to capture variations in heart rhythm and waveform morphology makes it possible to identify potentially life-threatening conditions such as myocardial infarction and atrial fibrillation, allowing timely interventions [3, 4].

ECG semantic segmentation occupies a distinct position within automated ECG analysis. While detection and classification approaches often focus on identifying events (e.g., R-peaks) or assigning beat/rhythm labels, semantic segmentation aims to delineate waveform components by labeling each time sample according to its physiological origin. This formulation provides a structured representation of the ECG morphology and naturally supports clinically relevant boundary estimation (onsets and offsets), which is essential for interval-based assessment and for building explainable pipelines that connect model outputs to recognizable electrophysiological phenomena.

Accurate segmentation is challenging because ECG morphology varies substantially across patients, leads, and recording conditions, and because the classes of interest are highly imbalanced: the background (isoline) constitutes most samples, whereas P-waves and parts of the T-wave can be low-amplitude and short in duration, making boundaries ambiguous even for experts. These factors can lead to systematic degradation in segmentation quality for the smallest classes, despite seemingly strong overall performance. Motivated by this, we investigate personalized adaptation to improve waveform delineation-especially for clinically important but harder-to-segment components-while retaining a unified segmentation framework and standardized evaluation that reflects both class-wise labeling quality and boundary accuracy.

The importance of ECG is underscored by its widespread use in emergencies and routine medical care. For instance, during acute myocardial infarction, an ECG can reveal critical changes in the ST segment, leading to decisions regarding reperfusion therapies that can significantly reduce mortality rates [5, 6]. On the other hand, during atrial fibrillation episodes, there is a noticeable absence of P waves and an irregularity in the duration of consecutive beats. In addition, an ECG is pivotal for long-term monitoring and continuous assessment of conditions such as heart failure and post-infarction arrhythmias in outpatient settings. The data provided by ECG enable clinicians to tailor treatment plans and monitor disease progression, thus improving patient outcomes [7, 8].

In addition, the simplicity, accessibility, and relatively low cost of the ECG contribute to its global application, particularly in resource-limited settings where advanced diagnostic tools may not be available. Its portability makes it ideal for use in remote areas and mobile health units, extending its reach and impact on public health [9].

In recent years, there has been a significant increase in studies (with millions of ECGs being analyzed) that employ artificial intelligence and automatic interpretation systems as additional tools for electrocardiography. Some results have demonstrated the ability of these new systems to identify and predict the onset of certain arrhythmias as well as outcomes such as strokes [10].

In general, automatic ECG analysis involves essential steps, namely, the preparation of raw ECG data through signal preprocessing and segmentation, feature extraction, and beat classification. ECG analysis is performed using computer-based methods, ranges from signal processing techniques that combine a set of rules to machine learning techniques when digital ECG databases are available for research. The method employed is either specific or hybrid (combining empirical and statistical techniques) [11], and the goal of the analysis involves either one particular step or all the necessary steps to provide a robust ECG signal analysis [12]. Andreão et al. [12] pioneered the use of a hybrid approach that combined wavelets and hidden Markov models (HMM) for a complete ECG analysis, including preprocessing, and segmentation of ECG signals.

Over the past few years, deep neural networks have shown promising results owing to the availability of ECG signal datasets and the high computer power to run deep learning algorithms. Lee et al. [13] employed convolutional neural networks (CNNs) to detect QRS complexes in capacitive ECG signals, addressing the challenges posed by signal noise and variations. The application of deep CNNs in this context has demonstrated significant improvements in detection accuracy, highlighting the potential of convolutional architectures for spatial feature extraction in ECG analysis.

Darmawahyuni et al. [14] further advanced this field by comparing the performances of recurrent neural networks (RNNs), specifically GRU and LSTM architectures, in detecting myocardial infarction using the PTB Diagnostic ECG database. Their study revealed that LSTM networks outperformed simpler architectures, with a sensitivity of 98.49% and a specificity of 97.97%. This finding underscores the advantages of long-short term memory networks in capturing the temporal dependencies inherent in ECG data. These studies confirmed the growing importance of deep neural networks in the automatic analysis of ECG signals, showcasing advancements in both recurrent and convolutional models.

However, most studies employing deep neural networks present some limitations in terms of lack of interpretability and transparency, as well as dependency on the ECG training dataset [15, 16]. Consequently, the necessary information on the precise localization of beat waveforms and their influence on the classification output is not available. There have been some attempts to meet the explainability requirement through techniques such as Grad-Cam [17], but these have failed to provide precise localization of the beat waveform.

A necessary and possible evolution of those approaches would be a hybrid deep neural network architecture which includes preprocessing, feature extraction, and segmentation of ECG signals [18].

One of the primary challenges is the complexity and variability inherent in ECG data as patients present varying heart morphologies. This heterogeneity poses difficulties in the generalization of models across diverse datasets. Moreover, noise and artifacts in ECG signals, such as baseline wander and electrical interference, often degrade the accuracy of segmentation algorithms, particularly in ambulatory or remote-monitoring scenarios [19, 20].

Another significant challenge is the scarcity of high-quality annotated ECG datasets. Although public databases, such as QT and MIT-BIH, exist, they are often limited in size and diversity. The lack of large, diverse, and well-annotated datasets hampers the training of robust deep neural networks. Transfer learning and data augmentation strategies attempt to mitigate this limitation. However, they cannot fully replace comprehensive data availability [21].

Explainability is also a critical issue in deep-learning-based ECG analysis. Although deep neural networks can achieve high accuracy, their decision-making processes are often seen as a ”black box,” making it difficult for clinicians to trust or verify the outputs. The lack of transparency in how these models segment and classify ECG signals can limit their clinical adoption because medical professionals typically require clear justifications for diagnoses derived from computational methods.

The primary objective of this study is to develop a hybrid deep neural network architecture to perform ECG processing and segmentation, ensuring that beat waveforms can be accurately detected and delineated within the ECG signals.

The limitations of current methods in dealing with heterogeneous data and the lack of generalization are addressed in this study through a patient adaptation training step, where complex ECG signals are selected to test a patient adaptation training strategy.

An important aspect of this study is the implementation of a decision-making layer that can generate comprehensive reports automatically, allowing healthcare professionals to better interpret the results.

The temporal information of the ECG signal segments was originally explored using the proposed algorithm. A new loss function was proposed to balance the need for accurate classification at each time step with the aim of smoothening temporal transitions and controlling sequence lengths.

Finally, the experimental protocol employs a publicly available ECG database called the QT Database, which contains annotated segments and classifications of cardiac events. The performance of the proposed architecture is compared to that of other works following the 2sCSE and AAMI standards [22, 23] to assess the effectiveness of this approach in clinical practice.

## Methods

The methodology employed in this study focuses on training a hybrid deep neural network architecture for the preprocessing, feature extraction, and segmentation of ECG signals with the aim of supporting decision-making algorithms in the diagnosis of cardiac episodes, such as atrial fibrillation, ventricular overload, and coronary heart disease. The selected dataset is the QT Database [11] containing beat waveform annotations made by cardiologists. These records undergo a data preparation stage, which includes filtering out noise and normalizing the ECG signals to ensure compatibility with the input range of the neural network. Each ECG record is then subdivided into time intervals while preserving its chronological order because temporal dependencies within the ECG data are important during the analysis.

The proposed hybrid deep neural network architecture comprises different topologies of deep neural networks including recurrent neural networks (RNNs), long short-term memory networks (LSTMs), and convolutional neural networks (CNNs). The networks are trained using labeled data which convey the time position of each beat waveform, namely the P-wave, QRS complex, and T-waves. Performance metrics such as sensitivity, specificity, and accuracy are tracked throughout the experiments to compare the proposed models with existing methods. A complete description of each step is presented in the following sections.

### Data

This study employed the QT database available on the PhysioNet website [24]. This database was selected because it was designed to address the scarcity of standardized databases for the development and assessment of computer methods devoted to automatic beat detection and segmentation. It includes a variety of ECG morphologies that are not limited to signals from healthy individuals. The database consists of 105 records, each 15 min long, recorded from two channels at a sampling rate of 250 Hz. For signals originally sampled at different frequencies, conversion to 250 Hz was performed wherever necessary. Expert cardiologists annotated wave occurrences for at least 30 s in each record.

To build and evaluate the proposed method, the database was divided as follows. i) Generic model approach: the subjects of the database were randomly split into training (80%) and testing (20%) subsets. This procedure was repeated five times each after a random distribution of the subjects to minimize dataset bias. ii) Personalized model approach: The QT database has two types of beat annotations: computer-based annotations (pu file) and cardiologist-based annotations (q1c file). These two types of annotations were compared, and then the F1-score was computed for each ECG record. The 15 records with the lowest F1-scores were selected as the test subset, whereas the remaining records formed the training subset. This dataset was designed to demonstrate the ability of the proposed method to enhance the performance through patient-specific model adaptation.

### Preprocessing

Pre-processing of the ECG signal involves several essential filtering procedures to ensure the accuracy and reliability of the data used for segmentation. ECG signals are often contaminated by various forms of noise, such as 60 Hz power line interference, baseline wander caused by respiration, and composite noise from other sources. To address these issues, filtering methods were applied, including a three-pole low-pass Butterworth filter with a cutoff frequency of 20 Hz. This empirically chosen configuration effectively removes high-frequency noise and preserves the integrity of the underlying ECG signal. Additionally, two median filters with sliding windows of 200 ms and 600 ms are employed to remove specific components of the ECG waveform. The 200 ms filter targets and removes the QRS complex and P waves, while the 600 ms filter eliminates T waves. These filters prevent the phase-shift problems that are common in conventional filters by utilizing zero-phase filtering from the SciPy library. After the filtering process, a normalization step is performed to address the amplitude variations present in the different ECG recordings. The signals are scaled to a range of −1 to +1, ensuring consistency across all datasets.

Finally, the ECG signal is normalized using the QRS peak amplitude as a reference. This normalization process employs an auxiliary algorithm based on the QRS method from the BioSPPy library [25]. This step identifies the most prominent R-wave peaks within the specified intervals, ensuring accurate peak detection and minimizing processing errors. The ECG signals are then normalized using the median value of the detected peaks. This robust normalization process preserves the original shape of the signal while compensating for outliers and amplitude fluctuations, resulting in a more reliable ECG signal for subsequent segmentation and classification tasks.

### Model architecture

The proposed deep learning architecture leverages a hybrid approach that combines CNNs and bidirectional BLSTM layers enriched with attention mechanisms. The model processes two separate one-dimensional input channels, each representing a 2000-sample ECG segment. The two input signals are first processed using independent CNN branches, which enabled the extraction of local morphological features. Each convolutional branch is followed by batch normalization layers to stabilize training and to improve generalization. After extracting the features from both input streams, the model concatenates these representations and merges the information into a single sequence. This joint feature representation is then processed by a bidirectional LSTM layer that captures the temporal dependencies in both the forward and backward directions. An attention mechanism is subsequently applied to help the model selectively focus on the most informative time steps. Finally, the model includes fully connected layers and a dropout layer before producing a sequence of class probabilities using the softmax output layer.

**Fig. 1:**
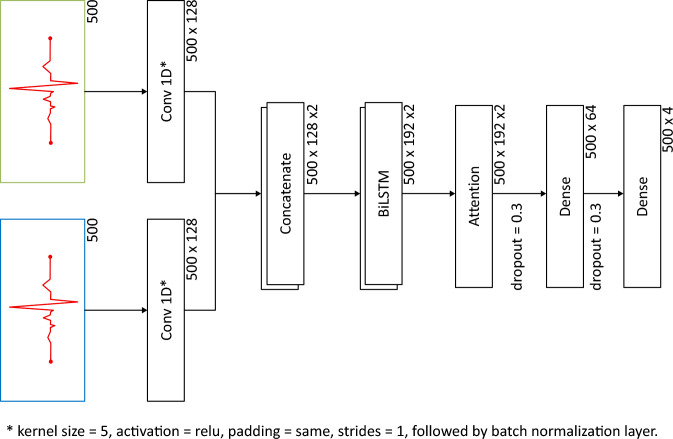
Overview of the proposed two-channel ECG semantic segmentation network. Each input branch receives a one-dimensional ECG segment (2 channels, 2000 samples), extracts local morphological features using stacked 1D convolutions, and merges representations before temporal modeling with a bidirectional LSTM. An attention mechanism emphasizes informative time steps for delineation. The network outputs, for each sample in the segment, a probability distribution over four semantic classes (background/isoline, P-wave, QRS complex, T-wave)

The key hyperparameters of the architecture, such as the number of convolutional filters, number of LSTM units, and dropout rate, are determined using a hyperparameter optimization process. A random search was employed to explore the hyperparameter space, sampling configurations over ten trials with one execution per trial, and comparing them based on the loss function values of the validation subset. The loss function is custom-defined for a specific segmentation task. Figure 1 provides an overview of the model architecture, including the input dimensions, layer configurations, and output specifications.

The final model is trained using the Adam optimizer and a custom loss function is designed for the segmentation task. Through patient-specific fine-tuning and hyperparameter optimization, this model aims to achieve a robust and accurate ECG waveform segmentation performance.

### Segmentation

The proposed segmentation process is based on the use of deep neural networks to automatically detect and classify different ECG waveform segments. Four distinct segments corresponding to the four output classes of the network are utilized for network training.

**Fig. 2:**
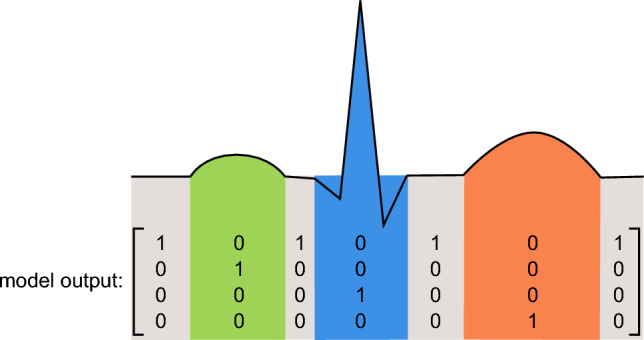
Sample-level semantic labeling used for training and evaluation. Each time sample is assigned one of four ECG semantic classes (background/isoline, P-wave, QRS complex, T-wave) using one-hot encoding derived from expert annotations. The illustration shows how contiguous samples belonging to a waveform (e.g., P-wave) form a labeled segment with clinically meaningful onset and offset boundaries

Waveform classes are defined using one-hot encoding based on annotations by expert cardiologist. This algorithm generates a binary matrix with four columns corresponding to the isoline, P-wave, QRS complex, and T wave, with each row representing an ECG sample, as illustrated in Fig. 2. This encoding is then used to train the neural network for accurate classification of the ECG components, which is essential for precise cardiac diagnosis.

For generic model training, the preprocessed ECG signal is divided into blocks of 500 samples without overlapping, which corresponded to a 2 s segment. These blocks are processed sequentially to preserve the temporal structure of the signal, which is a vital factor for effective analysis using recurrent neural networks (RNNs).

For the personalized model, 20% of the signal was used to fine-tune the generic model for each subject, and the remaining signal was used to test the performance of the method. Consequently, the personalized evaluation measures intra-patient adaptation - the ability of the model to improve on held-out data from the same patient after seeing a small labeled portion - rather than cross-patient generalization to entirely unseen subjects.

Finally, a post-processing step is carried out to validate the segmentation output as follows. A waveform is considered detected when it meets two specific criteria: (i) the onset of the detected wave occurs prior to the termination of the expert annotation, and (ii) the termination of the detected wave occurs after the onset of the expert annotation.

### Training

The proposed network training process is carried out in two main stages: (1) training a generic model using the entire training set and (2) adapting this generic model to individual patient data with only a small set of patient-specific annotations. This strategy is aimed at enhancing the segmentation performance of the model by introducing patient-level personalization with minimal manual annotation.

#### Generic Model Training

The proposed hybrid architecture is first trained using computer-based annotations provided by the QT database to produce a generic model. The database was randomly divided into three subsets: a training set with 80% of the subjects, a validation set with 10% of the subjects, and a test set with 10% of the subjects. The ECG signals were then divided into non-overlapping 500-sample (2 s) segments. From the resulting dataset, 10% of the samples were used as a validation set to ensure a proper evaluation of the model’s generalization capabilities during training.

A hyperparameter search is conducted to determine the optimal model configuration. The search space includes the number of 1D convolutional filters in the first two convolutional layers, number of units in the bi-directional LSTM layer, and dropout rate applied before the final output layer. A random search strategy is employed to explore the defined parameter ranges over five epochs. The set of hyperparameters that yields the best performance in the validation set is selected to define the final architecture of the generic model.

The generic model is then trained for 10 epochs using mini-batches of size 32. Early stopping is applied using 5 epochs patience, preventing overfitting and promoting convergence to a well-generalized solution.

#### Personalized Model Training

After obtaining the generic model, we seek to improve the performance of the model for a given patient by adapting it using a small proportion (20%) of the patient’s ECG data. The objective is to refine the model parameters such that the segmentation task aligns more closely with the patient’s specific signal characteristics, potentially capturing unique waveform patterns or artifacts present in the individual’s ECG.

To achieve this, a patient-specific adaptation subset is selected from 20% of the patient’s ECG signal, manually labelled by a cardiologist. A data augmentation process is implemented to increase the number of training segments available from the patient, thereby enhancing the adaptation. The adapted model is trained for 5 epochs using patient-specific samples, with 40% of the augmented data serving as the validation set. Early stopping with 3 epochs of patience is employed to ensure that the model does not overfit to the patient’s limited data.

By following this two-stage procedure, first learning a generalizable representation from the full training set and then adapting it to a specific patient, our method aims to balance the benefits of large-scale general training with the advantages of patient-specific fine-tuning for improved ECG segmentation performance.

### Proposed Loss Function

When modeling sequential data, it is often desirable to ensure accurate predictions at each time step and penalize undesirable behaviors, such as abrupt changes between predicted classes or unnecessarily long output sequences. To achieve these objectives, we propose a composite loss function that integrates the three main components namely the base loss term for classification accuracy, transition penalty to reduce unnatural temporal variations, and length penalty to control long sequences.

Formally, let $y_{\text {true}}(t)$ be the probabilities of the ground truth class at time t, and $y_{\text {pred}}(t)$ be the model’s predicted probabilities at time t, where t indexes the time steps of the sequence. We assume that the sequences have variable lengths, and that the model outputs a probability distribution over C classes at each time step.

#### Step 1: Base Loss Calculation

Base loss was computed using categorical cross-entropy. This loss measures the extent to which the predicted distribution of the model aligns with the true distribution at each timestep. For a single time step t, the base loss is defined as

$${\mathrm{base\_loss}}(t) = -\sum _{c=1}^{C} y_{\mathrm{true}, c}(t) \log (y_{\text {pred}, c}(t)).$$

Base loss is typically averaged over all the time steps across the entire sequence.

$${\mathrm{base\_loss}} = \frac{1}{T} \sum _{t=1}^{T} \left( -\sum _{c=1}^{C} y_{\text {true}, c}(t) \log (y_{\text {pred}, c}(t))\right) ,$$

where T is the sequence length.

***Example:***

If at time step t=0:

$$y_{\text {true}} = [1, 0, 0, 0] \quad (\text {True class: A})$$

$$y_{\text {pred}} = [0.9, 0.1, 0.0, 0.0]$$

Then:

$$\begin{aligned} \text {base}\_\text {loss}(0)= & -(1 \cdot \log (0.9) + 0 \cdot \log (0.1) + 0 \cdot \log (0.0) \\ & + 0 \cdot \log (0.0)) \approx -\log (0.9) \approx 0.105. \end{aligned}$$

#### Step 2: Transition Computation

To quantify the abrupt changes between consecutive time steps, we defined a transition measure as the absolute difference in the predicted probabilities between time steps t and t+1:

$$\text {transitions}(t \rightarrow t+1) = |y_{\text {pred}}(t+1) - y_{\text {pred}}(t)|.$$

***Example:*** If:

$$y_{\text {pred}}(2) = [0.8, 0.2, 0.0, 0.0], \quad y_{\text {pred}}(3) = [0.3, 0.6, 0.1, 0.0],$$

then:

$$|y_{\text {pred}}(3) - y_{\text {pred}}(2)| = [0.5, 0.4, 0.1, 0.0].$$

#### Step 3: Class Change Mask

Not all transitions are undesirable. Transitions are allowed when the true class changes. We define a binary mask that indicates whether the ground truth changes between time steps as follows:

$$\text {mask}(t \rightarrow t+1) = {\left\{ \begin{array}{ll} 1 & \text {if } y_{\text {true}}(t) \ne y_{\text {true}}(t+1), \\ 0 & \text {otherwise.} \end{array}\right. }$$

***Example:*** If:

$$y_{\text {true}}(2) = [1,0,0,0] \ (A), \quad y_{\text {true}}(3) = [0,1,0,0] \ (B),$$

then the true class changes from A to B, so:

$$\text {mask}(2 \rightarrow 3) = 1.$$

#### Step 4: Transition Penalty

The transition penalty was computed by applying a mask and averaging over the classes.

$$\begin{aligned} & \text {transition}\_\text {penalty}(t \rightarrow t+1) \\= & \frac{1}{C} \sum _{c=1}^{C} \left( \text {transitions}_{c}(t \rightarrow t+1) \cdot \text {mask}(t \rightarrow t+1)\right) . \end{aligned}$$

***Example:*** Using the previous transitions:

$$\text {transitions}(2 \rightarrow 3) = [0.5, 0.4, 0.1, 0.0], \quad \text {mask}(2 \rightarrow 3) = 1,$$

$$\text {transition}\_\text {penalty}(2 \rightarrow 3) = \frac{0.5 + 0.4 + 0.1 + 0.0}{4} = 0.25.$$

#### Step 5: Length Penalty

Therefore, long sequences may be undesirable. To control sequence length, we defined a length penalty. Let L be the maximum sequence length allowed and M the observed maximum sequence length. If the sequence exceeds L, then we compute the excess

$$\text {excess} = \max (0, M - L).$$

The length penalty is defined as the mean excess across the training batch:

$$\text {length}\_\text {penalty} = \text {mean}(\text {excess}).$$

***Example:*** If L=40 and M=50:

$$\text {excess} = \max (0, 50 - 40) = 10,$$

$$\text {length}\_\text {penalty} = 10.$$

#### Step 6: Combining the Losses

The total loss function is a weighted combination of base loss and penalties.

$$\text {total}\_\text {loss} = \text {base}\_\text {loss} + \alpha \cdot \text {transition}\_\text {penalty} + \beta \cdot \text {length}\_\text {penalty},$$

where α and β are hyperparameters controlling the relative importance of penalizing abrupt transitions and overly long sequences.

***Example:*** If:

$$\begin{aligned} & \text {base}\_\text {loss} = 0.105, \\ & \text {transition}\_\text {penalty} = 0.25, \\ & \text {length}\_\text {penalty} = 10, \end{aligned}$$

$$\alpha = 0.4, \quad \beta = 0.4,$$

then:

$$\text {total}\_\text {loss} = 0.105 + 0.4 \cdot 0.25 + 0.4 \cdot 10 = 0.105 + 0.1 + 4 = 4.205.$$

***Choice of ***α ***and ***β. The weighting parameters α and β were selected empirically based on preliminary experiments on the training/validation split, with the goal of balancing (i) stable optimization and (ii) improved boundary delineation for minority waveform classes without degrading QRS performance. In particular, we evaluated a small number of candidate settings and chose the final values that produced consistent validation improvements in wave-specific delineation (P- and T-wave onset/offset errors) while maintaining comparable overall segmentation quality. Although a full sensitivity analysis over α and β is beyond the scope of this work, we explicitly report this selection strategy and include it as a limitation, noting that future work will investigate the robustness of these hyperparameters under broader sweeps and across datasets.

#### Summary

The proposed loss function effectively balances the need for accurate classification at each time step with need for smooth temporal transitions and controlled sequence lengths. The base loss term encourages correct predictions, whereas the transition and length penalties guide the model to produce more temporally coherent and appropriately sized sequences. Together, these components lead to models that are not only accurate but also robust and suitable for practical applications in time-series segmentation and sequence prediction tasks.

### Post Processing

The post-processing phase focuses on refining the ECG signal outputs after segmentation to improve the quality and interpretability of the results. One of the key techniques applied is a smoothing algorithm that reduces abrupt variations between classified segments, thereby ensuring a more coherent interpretation of the cardiac cycle. This involves the application of a median filter to the classified labels, effectively minimizing noise and short-duration misclassifications that may have occurred during the earlier stages of processing.

Additionally, the system implements a correction mechanism to address the possible misdetection of key waveforms, such as the QRS complex or T-wave, by comparing the detected waveforms with physiological constraints (e.g., the typical duration of a QRS complex or T-wave). When inconsistencies are found, adjustments are made to realign the boundaries of the detected segments, thereby improving accuracy.

Furthermore, post-processing includes the generation of a confidence score for each detected class based on the softmax output of the network. This allows for better clinical interpretation because healthcare professionals can assess the reliability of predictions made by the automated system. The final ECG signal is presented in a manner that facilitates visual validation by experts, with clear demarcations of waveforms and associated confidence levels. This step ensures that the results are both clinically relevant and robust for practical application.

### Evaluation Metrics and Class Imbalance

We evaluate segmentation performance for each semantic class $c \in \{1,\dots ,C\}$ using standard confusion-matrix metrics computed at the sample level. Let $y_t \in \{1,\dots ,C\}$ denote the ground-truth label at time sample *t* and $\hat{y}_t$ the predicted label. For a given class *c*, we define:

1 $$\begin{aligned} TP_c&= \sum _t 1\{y_t=c \wedge \hat{y}_t=c\}, \nonumber \\ FP_c&= \sum _t 1\{y_t\ne c \wedge \hat{y}_t=c\}, \nonumber \\ FN_c&= \sum _t 1\{y_t=c \wedge \hat{y}_t\ne c\}, \nonumber \\ TN_c&= \sum _t 1\{y_t\ne c \wedge \hat{y}_t\ne c\}. \end{aligned}$$

From these quantities we compute sensitivity (recall), specificity, precision, and F1-score:

2 $$\begin{aligned} \textrm{Sensitivity}_c&= \frac{TP_c}{TP_c+FN_c}, \textrm{Specificity}_c = \frac{TN_c}{TN_c+FP_c}, \end{aligned}$$

3 $$\begin{aligned} \textrm{Precision}_c&= \frac{TP_c}{TP_c+FP_c}, \nonumber \\ \textrm{F1}_c&= \frac{2\cdot \textrm{Precision}_c \cdot \textrm{Sensitivity}_c}{\textrm{Precision}_c+\textrm{Sensitivity}_c}. \end{aligned}$$

To quantify delineation accuracy, we additionally report onset and offset errors for each wave class (P-wave, QRS complex, and T-wave). For each annotated waveform instance *k* of class *c*, let $\tau ^{(k)}_{\textrm{on},c}$ and $\tau ^{(k)}_{\textrm{off},c}$ be the ground-truth onset and offset sample indices, and $\hat{\tau }^{(k)}_{\textrm{on},c}$, $\hat{\tau }^{(k)}_{\textrm{off},c}$ the corresponding predicted boundary indices. We define signed boundary errors (in samples) as:

4 $$\begin{aligned} e^{(k)}_{\textrm{on},c}&= \hat{\tau }^{(k)}_{\textrm{on},c} - \tau ^{(k)}_{\textrm{on},c},&e^{(k)}_{\textrm{off},c}&= \hat{\tau }^{(k)}_{\textrm{off},c} - \tau ^{(k)}_{\textrm{off},c}. \end{aligned}$$

We summarize boundary accuracy using mean absolute error (MAE) and optionally median absolute error (robust to outliers):

5 $$\begin{aligned} \textrm{MAE}_{\textrm{on},c}&= \frac{1}{K_c}\sum _{k=1}^{K_c} \left| e^{(k)}_{\textrm{on},c}\right| ,&\textrm{MAE}_{\textrm{off},c}&= \frac{1}{K_c}\sum _{k=1}^{K_c} \left| e^{(k)}_{\textrm{off},c}\right| , \end{aligned}$$

where $K_c$ is the number of waveform instances of class *c*. When reporting errors in milliseconds, we multiply by the sampling period Δt (ms/sample).

***Aggregation and class imbalance.*** ECG semantic segmentation is strongly class-imbalanced: background samples dominate the signal, while P-waves (and parts of T-waves) occupy relatively few samples. Under such imbalance, overall (micro-averaged) metrics can be dominated by the background class and may overstate performance on minority waves. To reflect clinically relevant performance, we report *per-class* metrics for each semantic class and emphasize wave classes (P/QRS/T). When a single summary is needed, we use macro-averaging across classes,

6 $$\begin{aligned} \mathrm {Macro\text {-}F1} = \frac{1}{C}\sum _{c=1}^{C}\textrm{F1}_c, \end{aligned}$$

which weights each class equally and is less sensitive to class frequency than micro-averaging. Boundary errors are computed per record then averaged; uncertainty via record-level bootstrap, providing a complementary view focused on onset/offset delineation rather than sample-wise prevalence.

### Statistical Analysis and Confidence Intervals

To quantify uncertainty in boundary delineation while ensuring equal contribution from each record, we computed 95% confidence intervals (CIs) via nonparametric bootstrap at the *record level*. For each record *r* and each waveform/boundary condition (class × onset/offset), we first summarized the set of record-level errors within that record by three statistics: the signed mean error, the mean absolute error (MAE), and the median error.

Let $s_r$ denote one of these per-record summary statistics (e.g., per-record MAE) and let $\{s_r\}_{r=1}^{R}$ be the collection across the *R* records available for that condition. We then generated $B=10{,}000$ bootstrap resamples by sampling *R* records with replacement and recomputing the statistic of interest across records:

7 $$\begin{aligned} \widehat{T}^{(b)} = T\!\left( \{s^{(b)}_r\}_{r=1}^{R}\right) , \qquad b=1,\dots ,B, \end{aligned}$$

where T(·) denotes the mean across records for signed mean error and MAE, and the median across records for the median error. The 95% CI was computed using the percentile method:

8 $$\mathrm{CI}_{95}(T) = \left[Q_{0.025}\left(\{\widehat{T}^{(b)}\}\right),\; Q_{0.975}\left(\{\widehat{T}^{(b)}\}\right)\right].$$

This record-level resampling gives equal weight to each record/patient and avoids overweighting records that contain more annotated beats.

## Results

Table 1 presents a comprehensive comparison of the performance of the generic model before and after patient-specific fine-tuning. The first section of the table shows the overall detection metrics, including sensitivity, specificity, and F1-score. The results clearly indicate that the personalized model outperforms the generic model on held-out segments from the same patients used for fine-tuning, confirming that even a small amount of patient-specific annotation can substantially improve within-patient delineation accuracy. The F1-score increases significantly for all ECG waves, demonstrating that the personalized model is more accurate and reliable in identifying cardiac waves.

In addition, the table includes a detailed analysis of the segmentation accuracies of the P-, QRS-, and T-waves. For the P-waves, the sensitivity increased from 0.919 to 0.996 and the F1-score increased from 0.618 to 0.849 after fine-tuning. The improved performance was followed by a substantial reduction in both onset and offset detection errors. For the QRS complexes, the model maintained its performance with a slight improvement in the F1-score (from 0.961 to 0.968) and a notable decrease in the onset and offset errors. Finally, for T-waves, the detection performance also improved in terms of specificity (0.849–0.887) and F1-score (0.918–0.940), along with a significant reduction in the onset and offset errors.

The performance of the proposed model were compared with those of traditional methods proposed by Andreão et al. (HMM) [12], Laguna et al. [11], and Martínez et al. (Wavelet) [26], which serve as scientific baselines, as shown in Tables 2 and 3.

Overall, these results confirm that patient-specific adaptation, evaluated on held-out data from the same individual, leads to more precise ECG wave detection, fewer false detections, and improved accuracy and reduced standard deviation in wave onset and offset localization. These gains are consistent with the expectation that intra-patient variability is lower than inter-patient variability, allowing the model to specialize its representations when exposed to even a small amount of patient-specific data.

**Table 1:** Comparison of detection and segmentation performances between the generic model and the personalized model

		Generic model	Personalized model
P wave	Sensitivity	0.919	0.996
	Specificity	0.466	0.740
	F1 Score	0.618	0.849
	Onset Error Mean (std)	29.62 ms (23.07)	17.41 ms (19.87)
	Offset Error Mean (std)	35.98 ms (25.28)	11.37 ms (12.43)
QRS	Sensitivity	0.998	0.992
	Specificity	0.927	0.945
	F1 Score	0.961	0.968
	Onset Error Mean (std)	11.72 ms (9.34)	7.68 ms (7.10)
	Offset Error Mean (std)	23.24 (26.33)	10.59 (19.18)
T wave	Sensitivity	1.000	1.000
	Specificity	0.849	0.887
	F1 Score	0.918	0.940
	Onset Error Mean (std)	15.71 (17.17)	9.75 (17.59)
	Offset Error Mean (std)	65.46 (79.02)	27.13 (42.53)

QRS complex detection was analyzed in Table 3 because of its clinical relevance and widespread use in algorithm performance assessments. A small subset with the most difficult cases for the generic model was built to demonstrate the impact of the model adaptation procedure. Specific metrics such as true positives (TP), false positives (FP), and false negatives (FN) were calculated. Sensitivity (Se) is defined as the model’s ability to correctly detect QRS complexes in the annotations, whereas the positive predictive value (PP) assesses the proportion of correct detection relative to the to tal detection. The results demonstrated that the fine-tuned network achieves a higher efficacy, significantly reducing the FN and FP values.

**Table 2:** Waves detection performance comparison

Approach	Test set	Parameters	P	QRS	T
Generic model	Worst Cases	N Waves	223	483	462
		Se (%)	91.93	99.79	100.00
Personalized model	Worst Cases	N Waves	360	359	462
		Se (%)	99.55	99.17	100.00
Generic model	Test subset	N Waves	1177	1249	1271
		Se (%)	97.54	99.92	99.84
Personalized model	Test subset	N Waves	1177	1249	1271
		Se (%)	99.49	99.84	100.00
Londhe et al.	Test subset	N Waves	*	*	*
		Se (%)	88.66	94.05	90.50
Andreão et al.	Entire dataset	N Waves	3194	3623	3543
		Se (%)	98.65	99.92	99.94
Laguna et al.	Entire dataset	N Waves	3194	3623	3543
		Se (%)	97.70	99.92	99.77
Martínez et al.	Entire dataset	N Waves	3194	3623	3543
		Se (%)	98.87	99.97	99.00

* It was not possible to infer the number of beats from the paper

**Table 3:** QRS Detection performance comparison

Model	TP	FP	FN	Se (%)	PP (%)
Generic model (Worst)	482	38	1	99.80	92.70
Personalized model (Worst)	479	28	4	99.20	94.50
Generic model	1248	5	1	99.92	99.60
Personalized model	1247	6	2	99.84	99.52
Andreão et al	61468	258	75	99.88	99.58
Martínez et al	86824	107	68	99.92	99.88
Moody apud Martínez et al	84458	459	2434	97.20	99.46

## Discussion

The results presented in this study demonstrate the efficacy of deep neural networks in segmenting ECG signals. Under an intra-patient evaluation protocol - where the personalized model is fine-tuned on a small labeled portion of a patient’s recording and tested on the remaining held-out data from the same patient - the adapted model outperformed both the generic model and other approaches in the field, particularly in segmenting P and T waves. This underscores the importance of incorporating human annotations into machine learning models, as purely automated systems tend to underperform in complex scenarios, such as distinguishing between prominent T and R waves, low-amplitude P waves, and precise P- and T-wave boundary detection. However, a small reduction in the QRS complex detection performance indicates that further refinement is required. This may involve the use of more complex networks or additional data to handle the specific challenge of ECG signal segmentation in cases where the wave morphologies in a given patient change over time.

Generalizability of the models is another critical consideration. Given that the dataset had a limited number of annotated heartbeats, this study highlighted the potential limitations of scaling these models without adequate data. Large annotated datasets are vital for the robustness of deep learning approaches, especially when dealing with biomedical signals.

It is important to note that the evaluation of the personalized model reflects intra-patient adaptation rather than cross-patient generalization. Because fine-tuning and testing are performed on disjoint portions of the same patient’s recording, the reported improvements quantify the model’s capacity to learn individual morphological patterns - such as patient-specific P-wave amplitude, T-wave shape, and baseline characteristics - after observing a brief annotated segment from that same individual. This evaluation setting does not assess whether the adapted model transfers to entirely unseen patients without additional fine-tuning. However, this protocol aligns with a clinically realistic deployment scenario in which a patient undergoes longitudinal ECG monitoring: a clinician annotates a short initial segment (corresponding to the 20% used here), and the system subsequently provides improved automated delineation for the remainder of the recording and for future acquisitions from the same patient. In such a workflow, the cost of the initial annotation is amortized over extended monitoring periods, making the approach practical for applications such as Holter analysis, cardiac rehabilitation follow-up, and chronic arrhythmia surveillance. Future work should evaluate cross-patient generalization - for instance, by testing personalized models on subsequent recordings from the same patient acquired days or weeks apart - and investigate few-shot or meta-learning strategies that could reduce or eliminate the need for per-patient annotation while preserving the benefits of individualized adaptation.

When compared with other studies, the results of this research align with previous findings on the suitability of neural network topologies for ECG analysis. For instance, Darmawahyuni et al. [14] found that LSTM-based models outperformed simpler architectures for myocardial infarction detection, which is consistent with the improvements observed in this study through the use of LSTM and convolutional layers for segmentation tasks. Additionally, works by Andreão et al. [12] employing HMM also demonstrated the significance of expert annotations in improving model performance. Although HMM-based approaches have been developed, deep learning offers the ability to handle larger datasets with more complex signal variations, thus providing a more scalable solution.

Londhe and Atulkar [27] presented a deep-learning framework that fuses convolutional neural networks with BiLSTM layers for ECG wave segmentation, emphasizing the extraction of temporal dependencies alongside local morphological features. Their work demonstrated robust performance on the QT database, particularly by accurately delineating the P, QRS, and T waves in raw ECG signals. The study by Londhe et al. differs from other works in that it presents only the performance in terms of waveform detection. Consequently, we cannot comment about the precision of the waveform boundary segmentation, which has a direct influence on the detection accuracy. In contrast, our work extends beyond generic segmentation by introducing an individualized fine-tuning mechanism, thereby further improving the accuracy, especially for subtle waveform boundaries, such as those between P and T waves. Moreover, we adopted a custom loss function that encourages both accurate classification and smooth transitions, offering more refined segment delineation than the hybrid CNN-BiLSTM model.

Dissanayake and colleagues [28] employed a multi-stage temporal convolutional approach, leveraging varying dilation factors and a multi-level re finement process to segment one-dimensional (1D) biosignals (including ECG) and localize anomalies. In contrast, our approach places additional emphasis on patient-level customization. Although both approaches incorporate specialized loss functions to smooth the segmentation outputs, the personalized method integrates patient-specific data to retrain selected model parameters, further boosting segmentation precision. This adaptability, combined with the incorporation of an attention mechanism, allows the personalized model to handle intrapatient morphological variations more robustly than a purely TCN-based multistage pipeline.

This study contributes to the growing body of evidence that deep-learning architectures, particularly those combining recurrent and convolutional layers, are superior to traditional models in ECG signal processing. However, as noted in the recent literature, the interpretability of deep neural networks remains a challenge, and the need for transparent models that offer explanations is crucial for their adoption in clinical settings. We address this requirement by providing ECG segmentation as the network output. Consequently, beat waveforms and segments were identified for use as inputs for any classification model.

The findings of this study have significant implications for real-world applications, particularly for the development of clinical-grade tools for ECG analysis in longitudinal or patient-specific monitoring contexts. The ability of fine-tuned models to accurately detect key cardiac events such as P and T waves within a known patient’s recordings could lead to more reliable automated systems for continuous monitoring of conditions such as arrhythmias and post-infarction follow-up, where the same individual is monitored over extended periods and a brief initial expert annotation is feasible.

**Table 4:** Boundary error statistics (ms) with 95% bootstrap confidence intervals ($B=10{,}000$) computed by *record-level* resampling over per-record summary metrics (mean error and MAE). Signed mean error indicates systematic early/late bias, while MAE summarizes absolute boundary deviation. *N* is the number of records used for the corresponding estimate

Model	Wave	Boundary	*N*	Mean error (95% CI)	MAE (95% CI)
Generic	P	Onset	23	$7.01\,[3.36,\;11.06]$	$9.87\,[7.00,\;13.20]$
	P	Offset	23	$2.51\,[-4.48,\;8.85]$	$14.14\,[10.14,\;18.61]$
	QRS	Onset	36	$-3.96\,[-8.13,\;0.36]$	$10.74\,[8.02,\;13.58]$
	QRS	Offset	36	$1.92\,[-2.38,\;5.90]$	$10.17\,[7.52,\;13.14]$
	T	Onset	26	$-34.89\,[-53.45,\;-18.05]$	$40.27\,[23.82,\;58.77]$
	T	Offset	26	$38.91\,[22.56,\;60.60]$	$41.66\,[25.79,\;62.83]$
Personalized	P	Onset	23	$5.92\,[2.71,\;9.52]$	$8.39\,[5.77,\;11.39]$
	P	Offset	23	$2.16\,[-3.42,\;7.23]$	$10.25\,[6.92,\;13.97]$
	QRS	Onset	36	$-3.77\,[-7.78,\;0.36]$	$9.99\,[7.27,\;12.88]$
	QRS	Offset	36	$0.69\,[-3.44,\;4.57]$	$9.59\,[6.78,\;12.75]$
	T	Onset	26	$-38.25\,[-58.42,\;-20.26]$	$40.55\,[23.38,\;59.88]$
	T	Offset	26	$28.11\,[10.45,\;50.72]$	$35.17\,[19.35,\;56.43]$

As demonstrated in Table 4, personalization reduced MAE for P-wave boundaries and for QRS boundaries, and substantially improved T-wave offset delineation. Record-level CIs and paired nonparametric testing support these improvements, with the strongest effects found in P-wave offset and T-wave offset errors, as detailed in Table 5.

**Table 5:** Paired record-level comparison of MAE (Personalized − Generic) for boundary errors. Negative ΔMAE indicates improvement. Confidence intervals are obtained by bootstrap over records. The Wilcoxon signed-rank test is computed on paired per-record MAE values; $r_{\textrm{rb}}$ is the rank-biserial correlation effect size

Wave	Boundary	*N*	ΔMAE (95% CI)	*p* (Wilcoxon), $r_{\textrm{rb}}$
P	Onset	23	$-1.49\;[-2.67,\;-0.40]$	0.0166,-0.45
P	Offset	23	$-3.89\;[-6.46,\;-1.82]$	0.0007,-0.74
QRS	Onset	36	$-0.75\;[-1.21,\;-0.33]$	0.0014,-0.56
QRS	Offset	36	$-0.58\;[-1.54,\;0.45]$	0.0232,-0.53
T	Onset	26	$0.28\;[-1.52,\;2.51]$	0.0853,-0.43
T	Offset	26	$-6.48\;[-9.77,\;-2.83]$	0.0007,-0.63

In addition, the proposed model architecture favors the interpretability of the model’s decisions, which is vital for its adoption in clinical settings. In addition, incorporating expert feedback into the model refinement enhances the model’s ability to adapt to new and unseen data.

## Conclusions

This paper presents an original hybrid deep network architecture for automatic ECG signal analysis. The experimental results indicate that the personalized model, after fine-tuning with expert annotations, outperformed the generic model in detecting critical ECG waveforms under an intra-patient evaluation protocol, especially P- and T- waves, and achieved detection accuracies comparable to the state-of-the-art and onset and offset errors that met the CSE standards. This highlights the value of leveraging fine-tuning techniques and domain-specific networks to improve the precision and reliability of the ECG analysis.

This study contributes to the ongoing development of robust, accurate, and interpretable models for ECG signal analysis and provides insights into how deep learning can aid healthcare professionals in diagnosing cardiac abnormalities. By automating the segmentation of ECG signals using neural networks, this study enhanced the ability of the system to monitor heart conditions effectively, particularly in real-time clinical settings.

Based on the findings of this study, several recommendations are made for future research and practical applications. First, larger and more diverse datasets should be used to improve the generalizability of deep learning models in ECG analysis, addressing the limitations observed in QRS complex detection.

A limitation of this study is that the loss-weighting parameters (α, β) were selected empirically from a small set of candidates; future work will include a dedicated sensitivity analysis to quantify robustness across wider ranges and additional datasets.

In future work, a novel layer will be incorporated to classify ECG signals based on the output of the segmentation layer, thus offering greater interpretability for the model’s decisions. Furthermore, an active learning approach will be adopted to selectively identify the beats requiring annotation by experts, thereby maximizing the performance gains of the personalized model.

## Data Availability

The datasets used are publicly available and are mentioned in section Data.
